# Longitudinal Evidence for High-Level Patellar Tendon Strain as a Risk Factor for Tendinopathy in Adolescent Athletes

**DOI:** 10.1186/s40798-023-00627-y

**Published:** 2023-09-07

**Authors:** Falk Mersmann, Theresa Domroes, Meng-Shiuan Tsai, Nikolaos Pentidis, Arno Schroll, Sebastian Bohm, Adamantios Arampatzis

**Affiliations:** 1 Department of Training and Movement Sciences, Humboldt-Universität Zu Berlin, Unter Den Linden 6, 10099 Berlin, Germany; 2 Berlin School of Movement Science, Berlin, Germany

**Keywords:** Tendon overuse, Loading, Knee joint, Imbalances, Pathogenesis, Maturation, Youth, Training

## Abstract

**Background:**

High tendon strain leads to sub-rupture fatigue damage and net-catabolic signaling upon repetitive loading. While high levels of tendon strain occur in adolescent athletes at risk for tendinopathy, a direct association has not yet been established. Therefore, in this prospective longitudinal study, we examined the hypothesis that adolescent athletes who develop patellar tendon pain have shown increased levels of strain in advance.

**Methods:**

In 44 adolescent athletes (12–17 years old), patellar tendon mechanical properties were measured using ultrasonography and inverse dynamics at four time points during a season. Fourteen athletes developed clinically relevant tendon pain (SYM; i.e., reduction of the VISA-P score of at least 13 points), while 23 remained asymptomatic (ASYM; VISA-P score of > 87 points). Seven cases did not fall into one of these categories and were excluded. Tendon mechanical properties of SYM in the session before the development of symptoms were compared to a randomly selected session in ASYM.

**Results:**

Tendon strain was significantly higher in SYM compared to ASYM (*p* = 0.03). The risk ratio for developing symptoms was 2.3-fold higher in athletes with tendon strain ≥9% (*p* = 0.026). While there was no clear evidence for systematic differences of the force applied to the tendon or tendon stiffness between SYM and ASYM (*p* > 0.05), subgroup analysis indicated that tendon force increased prior to the development of symptoms only in SYM (*p* = 0.034).

**Discussio:**

The study provides novel longitudinal evidence that high tendon strain could be an important risk factor for patellar tendinopathy in adolescent athletes. We suggest that inadequate adaptation of tendon stiffness to increases in muscle strength may occur if adolescent athletes are subject to mechanical loading which does not  provide effective tendon stimulation.

## Background

Tendinopathy is a painful condition that commonly involves tenderness and swelling of the tendon and may cause severe functional impairments [1]. Affected tendons frequently show signs of degeneration, for example, a deterioration of the organization and structural integrity of the tissue, abnormal cellularity, increased levels of ground substance, and neovascularization [2]. The etiology of tendinopathy is complex and multifactorial, however, excessive loading and a disturbed metabolic response to loading are considered key factors in the pathogenesis of overuse tendinopathy [3–5]. Since the ultimate strain of a tendon is relatively constant [6], the magnitude of strain applied to a tendon is the main determinant of the mechanical demand placed upon the tissue during a loading cycle and determines both sub-rupture fatigue damage progression and the balance of anabolic-catabolic signaling upon repetitive loading [7–9]. In rabbit Achilles tendons, Wang and colleagues [8] found that cyclic application of 9% tendon strain deteriorates the structural integrity of the tissue and initiates a net-catabolic state. For this reason, it has been proposed that high levels of habitual tendon strain may predispose for tendon overuse injuries [10, 11].

Tendinopathy also affects the general population, but the prevalence is considerably higher in athletes compared to untrained individuals [12–14]. While the condition is rather uncommon in children, the prevalence increases during adolescence [15]. When investigating Achilles or patellar tendon strain during maximum voluntary contractions in vivo, we observed a distribution of high-level tendon strain in athletes and untrained peers from child- to adulthood that resembles the epidemiology of tendinopathy, i.e., the prevalence of high tendon strain was higher in athletes and seemed to increase with age [16–19]. Longitudinal observations in children [20], adolescents [19], and adults [21] suggest that an imbalance in the changes of the muscular force-generating capacity and the stiffness of the associated tendon results in marked fluctuations of tendon strain and episodes of elevated mechanical demand for the tendon. Adolescent athletes that demonstrated higher levels of strain further showed impairments of tendon micromorphology [22] that were also observed earlier in adult athletes with tendinopathy [23]. Since asymptomatic athletes with signs of tissue degeneration in ultrasound examinations are about four times more likely to develop symptoms [24], these findings reinforce the conception that high-level tendon strain may be an important risk factor for the development of tendinopathy.

Theoretically, muscle–tendon imbalances characterized by high tendon strain may develop if (a) an increase in muscle strength occurs without an appropriate adaptation of the tendon and/or (b) due to a mechanical weakening of the tendon as a consequence of overload or disturbed tissue homeostasis. In both cases, an increased level of strain may be an early indication that a tendon is at risk of developing a pathological state. Therefore, the purpose of the present longitudinal study was to prospectively investigate the role of tendon strain in the initiation of tendon pain. We regularly measured the patellar tendon mechanical properties of adolescent athletes from sports with a high prevalence of tendinopathy (i.e., handball, basketball, and volleyball) and examined the hypothesis that athletes who develop tendon pain—the leading symptom of tendinopathy—have shown higher levels of tendon strain than healthy athletes prior to becoming symptomatic. Considering that tendon strain and not force or stress is the best indicator of the mechanical demand on the tendon [22], we further hypothesized to observe no systematic differences in maximum tendon forces (i.e. the force applied to the tendon during a maximum voluntary contraction) but rather lower levels of tendon stiffness in the athletes that would become symptomatic.

## Methods

### Participants

A total of 66 healthy adolescent athletes (four female) from elite-level handball, basketball, and volleyball agreed to participate in the present study. The athletes were recruited on respective parents’ evenings during the preparatory phase of the season from five Berlin- (Germany) based teams (two male basketball, one male and one female volleyball, one male handball team) that participated in the highest national league. Inclusion criteria were 12–17 years of age and a weekly participation in sport-specific training of at least 8 hours. Exclusion criteria were musculoskeletal disorders of the lower extremities or neurological impairments. The participants and their legal guardians gave written informed consent to the experimental procedures, which were approved by the ethics committee of the Faculty of Humanities and Social Sciences, Humboldt-Universität zu Berlin (vote from 16.02.2018).

### Experimental Design

**Fig. 1:**
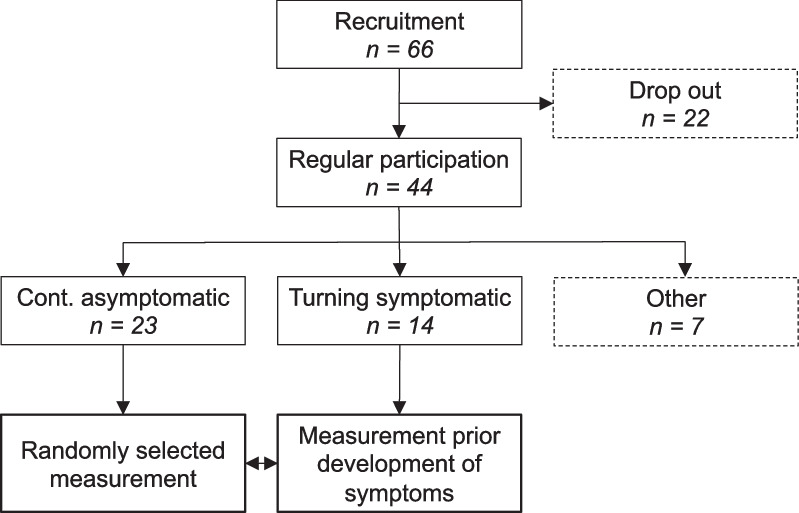
Flowchart illustrating the process from participant recruitment to the final data analysis. Regular participation refers to the attendance of at least two consecutive measurement sessions out of the four conducted over a competitive season. Athletes were considered continuously asymptomatic with VISA-P scores consistently above 87 points. A reduction of ≥ 13 points was considered “turning symptomatic” with a significant aggravation of symptoms. Other constellations (e.g., reporting less or equal to 87 points, yet with small stepwise reductions with each measurement) were excluded from the analysis

Baseline data of Quadriceps muscle strength and patellar tendon mechanical properties were assessed at the beginning of the competition phase (M1). The VISA-P questionnaire was used to confirm the absence of tendon pain and disability at baseline. An athlete was classified asymptomatic at a VISA-P score of  > 87 points, since the highest pain-free score is 100 and a deviation of 13 points is considered clinically relevant [25]. The average scores at baseline were 98 ± 3 points. Three follow-up assessments were scheduled in 10 to 15-week intervals (depending on the specific seasonal planning) midway in the competition phase (M2), at the end of the competition phase (M3), and in the subsequent preparation phase (M4). Twenty-two athletes were excluded as they did not attend at least two consecutive measurement sessions due to a lack of time (n = 15) or interest (n = 3), illness (n = 2) and injuries unrelated to the tendon (n = 2). From the remaining 44 athletes, 14 reported a clinically significant aggravation of symptoms of at least 13 VISA-P points during the season (5 in M2, 2 in M3, and 7 in M4). The VISA-P score was the single criterion for the assignment to the symptomatic group, as pain is the leading symptom in tendinopathy [26] and the questionnaire a validated tool for the assessment of related pain and disability [27], while e.g. structural abnormalities are not uncommon in asymptomatic athletes [28]. Since potentially other sources of pain may influence the responses to the questionnaire, the participants were asked to confirm that the pain originated from the patellar tendon and palpation was used as affirmation. The measurement prior to the development of symptoms was selected as the *session of interest* for the data analysis and the group is henceforth referred to as *symptomatic*. Twenty-three athletes were continuously asymptomatic, i.e., reporting VISA-P scores > 87 (on average 99 ± 2). The session of interest in this *asymptomatic* group was randomly selected using a random-number-generator. Seven participants did not fall into one of the two categories (e.g., reporting 87 or less points in the VISA-P questionnaire in a follow-up session, yet with a reduction of less than 13 points) and were excluded from further analysis (Fig. 1).

### Assessment of Quadriceps Muscle Strength

For the assessment of knee extensor strength, the participants performed maximum isometric knee extension contractions (MVC) on a dynamometer (Biodex Medical System 3, Shirley, NY, USA). The resultant joint moments were calculated using an established inverse dynamics approach [29] and electromyographic (EMG) data of the biceps femoris (long head) were used to estimate the contribution of the antagonistic muscles [30]. The necessary kinematic data were recorded using a Vicon motion capture system (V. 1.7.1; Vicon Motion Systems, Oxford, UK) with eight cameras operating at 250 Hz. The analog signals of the dynamometer and EMG data (Myon m320RX, Myon AG, Baar, CH) of the long head of the biceps femoris were captured at 1,000 Hz and transmitted to the Vicon system via an A-D converter.

After a standardized warm-up, the participants were seated and fixed on the dynamometer with a trunk angle of 85° (full hip extension equals 0°). Ten submaximal isometric contractions with increasing effort served as an additional warm-up, preconditioning of the tendon, and familiarization. Subsequently, three maximum contractions were performed at resting knee joint angles of 65°, 70°, and 75° (full knee extension equals 0°, values refer to the dynamometer data), as the approximate optimum angle for force generation is commonly reached from these starting positions. The resting period between trials was 3 min. An additional passive knee extension trial at 5 °/s was recorded with the shank of the participants fixed to the dynamometer lever pad to consider moments due to gravity. Finally, two trials of isometric knee flexion contractions with an EMG activity slightly below and above the activity that was registered during the knee extensions were recorded. The data were used to establish an activation-flexion moment relationship to estimate the knee flexion moments generated by the antagonists during the MVC trials based on the approach suggested for the ankle joint by Mademli and colleagues [30].

### Measurement of Patellar Tendon Mechanical Properties

Patellar tendon mechanical properties were derived from its force–elongation relationship, determined with a combination of inverse dynamics and ultrasound imaging. A 10-cm ultrasound probe (My Lab60; Esaote, Genova, IT; LA923, 7.5 MHz) was fixed over the longitudinal axis of the patellar tendon with a modified knee brace. Tendon elongation was captured during five trials of isometric ramp contractions in the individual joint position of the highest MVC trial. Guided by visual feedback on a monitor, the participants increased their force exertion from rest to maximum in about 5 s. To obtain the force applied to the patellar tendon, the knee extension moments were divided by the tendon moment arm, which was determined for each participant based on magnetic resonance images (MRI) acquired within one week before M1 [22 for details]. For M2, M3 and M4, the change in the individual moment arm was predicted by the changes in anthropometry [19]. In nine participants (3 symptomatic and 6 asymptomatic), it was not possible to schedule the MRI scanning due to time limitations. In these cases, the moment arm at baseline was predicted based on anthropometry as well. The change in the patellar tendon moment arm that occurs due to the inevitable rotation of the knee joint during isometric contractions was considered based on literature data [31]. The maximum tendon force (TF_max_) refers to the force applied to the tendon at the highest MVC trial of each participant. The elongation of the tendon during the ramp contractions was determined with a semi-automatic tracking of the deep insertion of the tendon at the patella and tibial tuberosity (Tracker Video Analysis and Modeling Tool V. 5.06, Open Source Physics, Aptos, CA, USA). Tendon slackness at rest was considered based on a spline fit through the deep insertion marks and two additional points along the lower border of the slack tendon [18]. Three co-authors contributed to the analysis of the US images (FM, TD & M-ST). While two were blinded, one (FM) had access to the personal data that belonged to the respective US images (incl. VISA-P scores) during analysis. However, the analysis was conducted prior to the next interview with the participants (which would reveal if an individual developed symptoms) and the tracking of the tendon origin and insertion was an automated software-driven process. The force–elongation relationship of the five trials of each participant was averaged to ensure excellent reliability [32]. As tendon forces generated during ramp contractions are usually lower compared to an MVC, tendon stiffness was calculated as slope of a linear regression between 50 and 80% TF_max_ (the latter representing a level of TF_max_ that was achieved by all participants in all five trials of ramp contractions). Maximum tendon elongation was then calculated by extrapolating this linear part of the force–elongation relationship (assessed during the ramp contractions) to the tendon force value of the highest MVC (i.e., TF_max_). The predicted maximum elongation was then normalized to the tendon rest length to obtain maximum tendon strain.

### Statistics

Frequentist statistics were conducted in SPSS (V. 26, IBM Corp., NY, USA), including a Shapiro–Wilk test for normal distribution and a student’s t-test for independent samples. Due to uncertainty considering the normal distribution of mass and tendon force, those parameters were analyzed with a Mann–Whitney-U test. Cohen’s *d* was calculated as an estimate of the effect size using the pooled standard deviation. Effect sizes of 0.2 ≤ *d* < 0.5 are referred to as small, 0.5 ≤ *d* < 0.8 as medium, and *d* ≥ 0.8 as large.

For the main outcome parameters (i.e., tendon force, stiffness, and strain), we additionally conducted a Bayesian analysis in R (RStudio V. 2022.07.1, RStudio Inc., MA, USA) using the *rstan* package to elucidate differences between the groups. The posterior distributions were calculated using two differently informed priors. The diffuse prior was a flat prior that was informed only considering the range of possible group means (μ) and standard deviations (σ) in both groups of μ = [0 kN, 10 kN] and σ = [0 kN, 5 kN] for tendon force, μ = [0 N/mm, 5000 N/mm] and σ = [0 N/mm, 2500 N/mm] for tendon stiffness, and μ = [0%, 20%] and σ = [0%, 10%] for strain. The informed prior was defined based on data from our earlier studies [18, 22, 33, 34] and the following means and standard error of mean as measure of uncertainty were assumed for the asymptomatic and symptomatic group, respectively: tendon force 3.98 ± 0.42 kN and 4.42 ± 0.42 kN, tendon stiffness 1192 ± 184 N/mm and 1112 ± 184 N/mm, strain 7.84 ± 0.89% and 9.50 ± 0.89%.

We further calculated the risk ratio for the development of symptoms based on tendon strain using the incidence of tendon pain in athletes that reached either common (< 9%) or high levels of tendon strain (≥ 9%) [10]. The alpha level for all statistical tests was set to 0.05.

To gain more insight into the changes of the mechanical properties of the tendon, we performed a sub-group analysis with the data from symptomatic athletes who did not report symptoms in the first two or three measurement sessions (n = 8). In those cases, we were able to analyze the changes of tendon strain, force, and stiffness from the preceding session to the *session of interest* (and compare the changes to the respective data of the asymptomatic group, n = 13) using a repeated measures ANOVA.

## Results

There was no significant difference between the asymptomatic and symptomatic group considering age (*p* = 0.79, *d* = 0.14), body height (*p* = 0.24, *d* = 0.40)or body mass of the athletes (*p* = 0.12, *d* = 0.54; Table 1).

**Table 1:** Anthropometric data of the asymptomatic and symptomatic athletes (prior to symptom development)

	Asymptomatic	Symptomatic	*p* value	Cohen’s *d*
	*n* = 23	*n* = 14		
Age (years)	15.0 ± 1.1	15.2 ± 1.8	0.79	0.14
Height (cm)	182.5 ± 10.4	186.9 ± 11.7	0.24	0.40
Mass (kg)	68.9 ± 11.6	75.7 ± 14.0	0.12	0.54

**Fig. 2:**
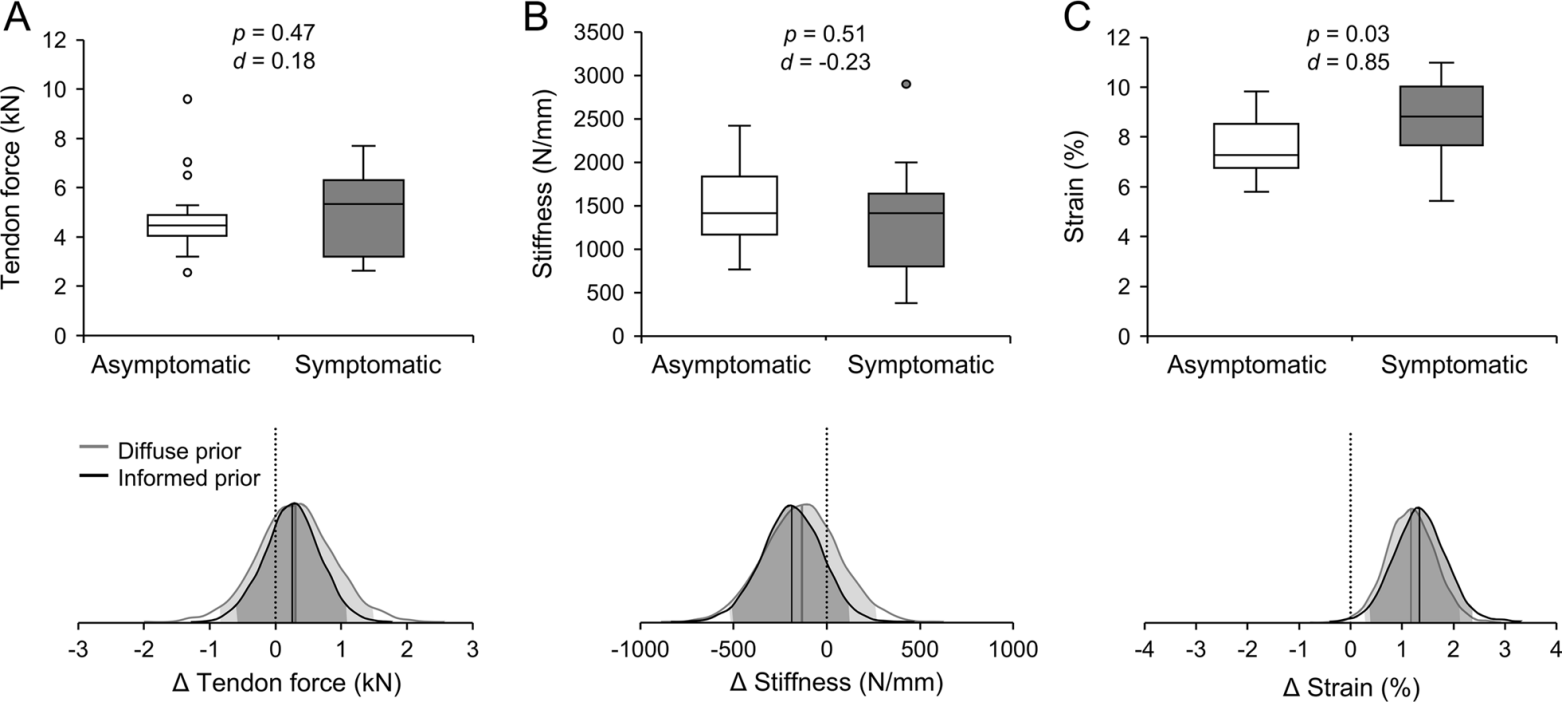
Force applied to the patellar tendon (**A**), tendon stiffness (**B**) and strain (**C**) in the asymptomatic (n = 23) and symptomatic group (prior to the development of symptoms; n = 14). Data points are shown as outliers if the distance to the 1^st^ or 3^rd^ quartile exceeded 1.5 times the interquartile range. The *p* values for independent sample testing  and Cohen’s *d* are given as well. The bottom panels show the posterior distributions of the differences between the two groups based on the diffuse (gray) or informed prior (black). A shift towards positive values indicates higher values in the symptomatic group. The 95% credibility interval is indicated as shaded areas in the graphs. The y-axes (not shown) of the probability density curves have an arbitrary unit with the integral of the curves being 1.

Tendon strain was significantly higher in the symptomatic athletes with a large effect size (*p* = 0.03, *d* = 0.85), indicating an increased mechanical demand on the patellar tendons of those athletes that were to develop symptoms (Fig. 2). Our Bayesian modeling suggests that the difference in tendon strain between future symptomatic and healthy athletes is most likely in the order of 1.2 to 1.4 percentage points of strain, based on the diffuse and informed prior, respectively. The probability of a difference of at least 0.5 percentage points is estimated at 91% and 95%, respectively (Fig. 2). Further, the risk ratio for developing symptoms was 2.3-fold higher in athletes  with tendon strain  ≥ 9% compared to athletes with tendon strains lower than 9% (*p* = 0.026; 95% confidence interval 1.1 to 4.9).

There was no significant difference to asymptomatic athletes considering tendon force (*p* = 0.47, *d* = 0.18) and stiffness (*p* = 0.51, *d* = − 0.23; Fig. 2), despite a small-sized effect on stiffness. When using the informed prior, the estimated probability that tendon stiffness in future symptomatic athletes is at least 150 N/mm (i.e., ~ 10%) less than in healthy ones was around 59% (Fig. 2). Both, the absolute knee extension moments (*p* = 0.42, *d* = 0.27) and the moments normalized to body mass (*p* = 0.71, *d* = − 0.13) were not significantly different between groups. There was a tendency towards a greater tendon moment arm in the symptomatic group with a medium-sized effect (*p* = 0.084; *d* = 0.60), while no significant differences of rest length were found (*p* = 0.53, *d* = 0.22; Table 2).

**Table 2:** Maximum voluntary isometric knee extension moment, moment normalized to body mass (norm), patellar tendon moment arm and rest length of the asymptomatic and symptomatic athletes (prior to symptom development)

	Asymptomatic	Symptomatic	*p* value	Cohen’s *d*
	*n* = 23	*n* = 14		
Moment (Nm)	259 ± 93	286 ± 105	0.42	0.27
Moment_norm_ (Nm/kg)	3.8 ± 0.9	3.7 ± 0.9	0.71	− 0.13
Tendon moment arm (mm)	54.2 ± 2.4	55.7 ± 2.5	0.08	0.60
Tendon rest length (mm)	52.7 ± 5.2	53.8 ± 5.5	0.53	0.22

**Fig. 3:**
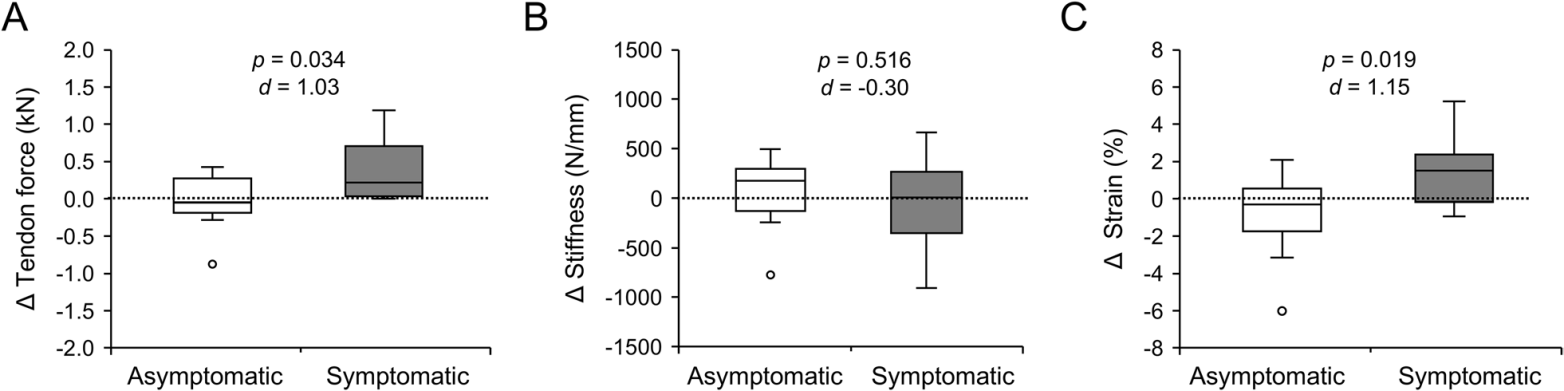
Changes in force applied to the patellar tendon (**A**), tendon stiffness (**B**) and strain (**C**) in a sub-group of the asymptomatic (n = 13) and symptomatic group (n = 8) from the preceding session to the last measurement session prior to symptom development in the affected athletes. Data points are shown as outliers if the distance to the 1st or 3rd quartile exceeded 1.5 times the interquartile range. The *p* values for independent sample testing as well as Cohen’s d are given as well.

In the sub-group analysis of the within-group changes from the preceding session to the *session of interest* (i.e., last session before symptom development), a time-by-group interaction was observed on tendon force (*p* = 0.034) and strain (*p* = 0.019), and a post-hoc comparison of the changes between time points suggests a greater increase of force in the symptomatic group (Fig. 3), while no significant differences were found in tendon stiffness, which remained relatively constant in both groups (main effect of time: *p* = 0.95, interaction: *p* = 0.52).

## Discussion

In the present prospective longitudinal study, we regularly measured the patellar tendon mechanical properties of adolescent athletes over a competitive season and provided first-time evidence that athletes who develop tendon pain and pain-related disability demonstrate higher levels of strain in advance compared to continuously asymptomatic athletes. The risk ratio for developing symptoms was 2.3-fold higher in athletes  with tendon strain  ≥ 9% compared to athletes with lower tendon strains. Neither tendon force nor tendon stiffness were significantly different between groups, yet a sub-group analysis indicated that an imbalanced increase in the force applied to the tendon rather than a reduction of stiffness was the reason for the elevated level of tendon strain.

The Bayesian modeling of our tendon strain data suggests that athletes who develop symptoms show with a probability of 95% strain values that are at least 0.5 percentage points higher than in healthy peers. That difference is of a magnitude that implies a severe increase of the mechanical demand on the tendon. In the experiments of Wren et al. [35], an increase of 0.5 percentage points in the initial strain of cyclically loaded human Achilles tendon specimen reduced the number of cycles until failure to about half. In vitro failure testing of tendons may not reflect the pathogenesis of tendinopathy in vivo. However, the magnitude of tendon strain also mediates sub-rupture fatigue damage, which includes collagen molecular denaturation, fibrillar kinking, recoil or rupture, and cell–matrix disruptions [7, 36]. The resultant loss of the mechanical integrity of the tissue may induce abnormal tenocyte stimulation and could contribute to the disturbed tissue homeostasis associated with tendinopathy [4, 37]. Also, Wang and colleagues [8] observed a net- catabolic tissue state and fatigue damage following cyclic stimulation of rabbit Achilles tendons when the applied strain was as high as 9%. When using this level of tendon strain as a criterion to group athletes with common and high-level tendon strain, we found a significant 2.3-fold  higher relative risk to develop tendon pain in the group with high-level strain. Although this threshold is based on animal data and, from a physiological perspective, there is probably rather a transition band towards levels of strain with potentially harmful consequences for the tissue, our findings suggest that it may be possible to identify athletes at risk for developing tendon pain using this threshold.

High levels of tendon strain have been observed earlier in risk groups for tendinopathy [17–19] as well as in patients with chronic Achilles tendinopathy [38, 39]. While lower levels of tendon stiffness in patients with patellar tendinopathy compared to healthy controls were reported by some [40, 41], yet not all studies [42–44], tendon strain has not been shown to be higher, as one might expect with regard to the results of the current study. Both methodological issues as well as physiological reasons might explain the discrepancy between our observations and those from others examining patients with tendinopathy [40–44]. None of these studies considered the individual variation in the knee joint angles where the highest joint moments can be generated, which—according to data from Sharifnezhad et al. [45]—can show interindividual differences of up to 20° (SD 6.5°). Further, all studies used an ultrasound array insufficient in length to capture the full length of the patellar tendon, which can be associated with a considerable error when measuring patellar tendon elongation during isometric contractions [46, 47]. However, more importantly, the isometric force exerted by the muscle at a given length—and thus the strain of the tendon—depends on the level of muscle activation. In healthy individuals, the activation level during isometric contractions is commonly very high [48], but pain during knee extension contractions due to tendinopathy reduces the level of muscle activation [49, 50]. While this may be different for the Achilles tendon [51], maximum patellar tendon strain measured in symptomatic individuals is unlikely representative for the level of strain the tendon was subjected to prior to the development of symptoms. The magnitude of patellar tendon strain observed in the present study  was comparatively high. However, just recently we started to consider the discrepancy of maximum tendon force during ramp contractions and an  actual MVC, which explains the slightly higher values compared to earlier reports [17–19]. The higher values compared with other groups (e.g., [46]) are most likely related to additional methodological aspects as mentioned above, particularly the reduced force potential of the knee extensors at 90° of knee flexion in most other studies.

In line with our hypothesis, maximum tendon force was not notably higher in the symptomatic group compared to the asymptomatic controls. Thus, a high muscle strength capacity per se is unlikely the reason for the increased levels of tendon strain observed in the symptomatic group. There was a small effect on tendon stiffness and an 88% probability (based on the Bayesian modeling using the informed prior) for lower values in the future symptomatic athletes. Yet, the difference between groups was not significant and the probability that the differences between groups were greater than 150 N/mm (i.e., ~ 10%; approximate measurement error, [see 52 for a discussion]) was only 59%. Though it cannot be excluded that the present study was underpowered to show small differences in stiffness, the results of the sub-group analysis indicate that an increase in the force that acts on the tendon which is not matched by an increase of tendon stiffness is the reason for the increase of tendon strain rather than a reduction of stiffness. This would also be in line with earlier observations indicating that fluctuations of tendon strain in athletes relate to changes in tendon force rather than stiffness [19–21] and that early stages of tendinopathy do not seem to be linked to an impairment of tendon mechanical properties [53]. It seems that some individuals show a reduced plasticity of the tendon to the sport-specific loading and develop an imbalance of muscle strength and tendon stiffness, as indicated by the high levels of tendon strain. Recently, Passini and colleagues [54] demonstrated interindividual differences in the activity of PIEZO1—a mechanosensitive ion channel—which influences the adaptation of tendons to mechanical loading and the degree of tendon stiffness reached in a given mechanical environment. Heinemeier et al. [55] further provided evidence that tendinopathy is preceded by a distorted tendon tissue turnover, which may also contribute to reducing the degree to which tendons adapt to the strength capacity of their muscle. Individuals that show an  attenuated adaptation of the tendon could be particularly predisposed for developing muscle–tendon imbalances and tendon overuse symptoms.

There are some limitations to this study that warrant discussion. The categorization of symptomatic athletes and asymptomatic athletes was based solely on the VISA-P questionnaire, which is a validated tool to assess tendon pain and pain-related disability in patients with tendinopathy. Though the athletes were asked to confirm the site of pain being the tendon, the location is no specific item in the questionnaire and, theoretically, a bias of the score due to other sources of pain at the knee is possible. As pain is the leading symptom in tendinopathy and structural abnormalities are not uncommon in asymptomatic athletes, no other diagnostic measures were used as classification criteria. Therefore, we are not able to differentiate how many athletes in both the symptomatic and asymptomatic group had indications  of structural changes of the tendon. However, the association of tendon strain or tendon pain development with structural changes of the tissue has been examined earlier [22, 24]. Finally, it should be noted that the sample size of the current study has been rather small, particularly considering the sub-group analysis of the intra-individual change in tendon strain. A simplification of tendon strain assessment may allow future studies to confirm our findings in a larger sample.

## Conclusion

In conclusion, the present prospective longitudinal study provides novel evidence that high-level tendon strain could be an important risk factor for the development of tendon pain in adolescent athletes. The optimization and implementation of techniques to quantify tendon strain in vivo could enable the identification of individuals with muscle–tendon imbalances and a potentially increased risk for the development of tendon pain [10]. Using 9% of tendon strain as a threshold, the data of the present study suggests that athletes reaching such high levels of tendon strain during MVCs have a 2.3-fold increased risk of developing tendon pain, though more data is needed to confirm this estimate. Further, our findings suggest that the increase of strain preceding the development of tendon pain is due to an imbalanced adaptation of tendon stiffness to increases in muscle strength. This may occur if adolescent athletes with certain preconditions—e.g., a distorted tissue turnover [55] or reduced mechanosensitivity [54]—act in a mechanical environment that does not  provide effective tendon adaptation stimulation. Integrating specific and more adequate stimuli for the tendon into the loading regimen of athletes, however, may induce beneficial effects on tendon metabolism and, given sufficient anabolic signaling, may promote tendon stiffness adaptation [33, 34]. While it remains unclear to what extent these findings may be limited to adolescent athletes,  they provide new opportunities for the prevention of tendon overuse injuries.

## Data Availability

The datasets generated during and/or analysed during the current study are available from the corresponding author on reasonable request.

## Abbreviations

ANOVA: Analysis of variance
ASYM: Asymptomatic athletes
EMG: Electromyography/electromyographic
M1…4: Measurement 1…4
MRI: Magnetic resonance imaging/images
MVC: Maximum voluntary contraction
SD: Standard deviation
SYM: Symptomatic athletes
TF_max_: Maximum force applied to the tendon
VISA-P: Victorian Insitute of Sports Assessment-Patellar
